# Organizing across disciplines to tackle shared computational challenges

**DOI:** 10.1016/j.patter.2026.101518

**Published:** 2026-04-10

**Authors:** Wojtek Treyde, Aleksy Kwiatkowski, Jascha Achterberg, Danyal Akarca, Martin Buttenschoen, Rory T. Byrne, Kieran Didi, Kristina Kordova, Jakub Lála, Jonathon Langford, Austin M. Mroz, Puria Radmard, Filip T. Szczypiński, Eva Sevenster, Inga Van den Bossche, Michał Wójcik

**Affiliations:** 1 The CompMotifs Initiative; 2 Chemistry Research Laboratory, University of Oxford, Oxford, UK; 3 Centre for Neural Circuits and Behaviour, University of Oxford, Oxford, UK; 4 Department of Electrical and Electronic Engineering, Imperial College London, London, UK; 5 MRC Cognition and Brain Sciences Unit, University of Cambridge, Cambridge, UK; 6 Department of Statistics, University of Oxford, Oxford, UK; 7 Department of Engineering, University of Cambridge, Cambridge, UK; 8 Department of Computer Science, University of Oxford, Oxford, UK; 9 Department of Veterinary Medicine, University of Cambridge, Cambridge, UK; 10 Department of Materials, Imperial College London, London, UK; 11 Department of Physics, Imperial College London, London, UK; 12 I-X Centre for AI in Science, Imperial College London, London, UK; 13 Department of Chemistry, Imperial College London, London, UK; 14 Department of Chemistry, Durham University, Durham, UK; 15 Department of Engineering Mathematics, University of Bristol, Bristol, UK; 16 Department of Physiology, Anatomy and Genetics, University of Oxford, Oxford, UK; 17 Department of Engineering Science, University of Oxford, Oxford, UK; 18 Kavli Institute for Nanoscience Discovery, University of Oxford, Oxford, UK

## Abstract

The Science through Computation Initiative unites sixteen early-career computational scientists spanning physics, biology, chemistry, and neuroscience. Over nine months of workshops and hackathons, they agreed on “computational motifs,” fundamental challenges that recur across their scientific disciplines, built prototype tools tackling them, and distilled actionable recommendations for the wider research community.

## Main text

Advances in computational power and the recent surge in the use of artificial intelligence (AI) across the sciences present an extraordinary opportunity for accelerating scientific discovery through computational techniques. We believe that many associated challenges are shared across scientific disciplines and that the most impactful tools to tackle them will emerge through interdisciplinary collaboration. To test this, we established the Science through Computation Initiative (CompMotifs), a group of 16 early-career computational scientists based in the UK, spanning physics, biology, chemistry, and neuroscience. Over 9 months, we convened workshops and hackathons and agreed on shared challenges and potential computational solutions.

This opinion details the challenges, which we call “computational motifs,” that we agreed are of great importance across our fields. Addressing them therefore has the potential for greater impact across all scientific domains. We aim to demonstrate that bringing young scientists together across disciplines, through discussion groups and hackathons, can produce prototype solutions. This opinion is also a call to action: we encourage others to build similar communities and tackle these challenges with us. We first introduce the organization of our workshops and hackathons, then present the computational motifs we discussed as well as hackathon prototypes and ideas for addressing them, and conclude by offering four actionable recommendations for the scientific community.

To explore different computational motifs, our initial working group met for monthly workshops to share pain points in our respective fields. We invited expert speakers who would begin the day with an outline of their state-of-the-art work. In the afternoons, we would discuss how this could be applied across our different fields and began working on prototypes. This process informed the design of our hackathons.

Our hackathons in San Francisco (40 places) and London (80 places) were oversubscribed, with 150 sign-ups, providing evidence of widespread enthusiasm for tackling scientific problems using computation. Our participants were recruited through social media and personal networks of external sponsors. We noticed early on that the initial recruitment attracted disproportionately few people identifying as women and recognized how significantly the wording of event advertisements can impact applicant demographics. Using tools such as the Gender Decoder,^1^ we revised our event description, resulting in a doubling of participants who identified as women. During the hackathons, we strongly encouraged teams to share ideas and assist one another. As organizers, we had significant power to set the tone and expectations, aiming to ensure a balance between collaboration and competition. Projects were judged on scientific impact and novelty, cross-disciplinary appeal, technical execution (prioritizing working prototypes over a polished interface), and presentation clarity. The focus throughout was on building community and solving problems of scientific value, not commercial ventures. Alongside expert judging, we held a community vote in which all participants selected which tools they would most want to use in their own research as a measure of adoption potential.

The computational motifs explored in the hackathons and workshops can be split into the two primary domains of the scientific process: *exploration* (how to decide which scientific problem to investigate) and *experimentation* (how to evaluate hypotheses). Exploration can be further divided into *social* challenges, which concern human behavior and interaction, and *conceptual* challenges, which concern how we think about or represent scientific knowledge. Most of these challenges are known,^2^^,^^3^ but we summarize them here and outline the proposed solutions that arose from our hackathons. Survey results collected during our hackathons show that applicants agreed with our computational motifs.^4^ A full list of projects and the challenges they aim to address is provided in Table 1.

**Table 1:** Overview of hackathon projects and concepts to tackle the shared challenges discussed in this opinion

Name	Type	Description
**Exploration: Social**		

Resonance.ai	prototype	Different disciplines use distinct terminologies for identical concepts, hindering recognition of shared problems. This chatbot matches conference attendees with relevant peers across disciplinary boundaries.
LikeMinds	prototype	Researchers often fail to discover potential collaborators outside their own field. This tool identifies shared scientific interests from social media (Bluesky) activity (likes/comments/shares) to suggest cross-disciplinary matches.
ELIE	prototype	Differing expectations and knowledge gaps between disciplines hinder productive interdisciplinary collaboration. ELIE maps a user’s existing knowledge against a target concept, then generates a tailored explanation addressing only their actual gaps.
Chippy	prototype	Research time is wasted on tedious nonscientific tasks, such as navigating fragmented data formats. This tool converts poorly formatted patent literature into structured, tabular, experimental data.
DeepCritic	prototype	Academic incentive structures discourage high-risk cross-disciplinary work, where early feedback is scarce. This tool provides an ensemble of LLM peer reviewers that offers early-stage feedback.
Incentivising Failure Publication	concept	Reluctance to share negative results leads to duplicated efforts, selection bias, and publication bias. We propose a platform where researchers earn tokens for submitting null results and failed experiments, redeemable for access to others’ datasets.

**Exploration: Conceptual**		

Latent Science	prototype	Without a shared language across disciplines, nonobvious connections between fields go unrecognized. This web app aims to map research publications into cross-disciplinary embeddings, revealing conceptual links between seemingly unrelated papers.

**Experimentation**		

DataRaccoon	prototype	Models often fail to generalize outside their training domain, partly due to poor data quality. DataRaccoon scans datasets using universal metrics to score quality and flag problematic variables or observations. The aim is to provide a data quality score to be published alongside conclusions made.
Fossilised LLMs	concept	It is difficult to evaluate whether AI tools can genuinely discover new phenomena. Fossilized LLMs aims to benchmark AI discovery by training models on historic data and testing whether they can predict known subsequent breakthroughs.
Protocompare	prototype	Many protocols exist for the same procedure, but researchers lack a systematic way to compare them. Protocompare aggregates protocols, breaks them into comparable steps, and ranks them by field-relevant metrics such as safety, cost, and computational expense.
SupplyScout	prototype	Inefficient procurement of lab materials slows experimental validation. SupplyScout uses a graph-based algorithm to optimize lab material sourcing and rank vendors.
Advanced Sensor Platforms	concept	The mismatch between computational and laboratory timescales makes it difficult to measure real-world performance and to provide rich datasets at scale. High-throughput experimentation, better laboratory model systems, and better sensors would provide richer, faster experimental readouts.
Cross-Disciplinary Fellowships	concept	Practical and cultural gaps persist between computational and experimental researchers. Fellowships placing computational scientists in wet labs and vice versa would build mutual understanding and close working-practice gaps.
ForGit About It!	prototype	Inconsistent version control undermines computational reproducibility. This tool monitors a codebase and auto-commits when a logical unit of work is complete, then generates an end-of-day progress summary.
GreatProtocols	prototype	Crucial experimental details are often lost, hampering reproducibility. This tool prompts researchers to capture missing metadata, then regenerates protocols tailored to the reader’s experience level.

Archival versions of the GitHub repositories are available in a Zenodo repository.^5^

In exploration, several social challenges resonated across all of our fields. First, different disciplines use distinct terminologies for identical concepts, which hinders recognition of shared problems (tackled by Resonance.ai, LikeMinds*,* and ELIE; Table 1). Second, a significant amount of research time is wasted on tedious nonscientific tasks, such as navigating fragmented software ecosystems, formatting data, and writing administrative text, much of which could be shared or standardized across fields (Chippy; Table 1). Third, academic incentive structures, with their “publish or perish” mentality, may discourage ambitious cross-disciplinary work^6^ (DeepCritic; Table 1). Fourth, progress across our fields is hindered by a reluctance to share data.^7^ In particular, existing reward structures and norms discourage the publication of negative results,^8^ leading to duplicated efforts, as well as selection bias (researchers choosing experiments they believe will work) and publication bias (researchers largely publishing positive results; Incentivising Failure Publication; Table 1). Finally, a further social hurdle arises from differing expectations around computation in interdisciplinary collaborations. Experimentalists may dismiss computational predictions if they are not “perfectly” predictive or if the underlying theory is insufficient. However, we argue that computational tools should be viewed as partners that reduce the overall rate of failure rather than prophets that eliminate it entirely.

The primary conceptual challenge that cut across our disciplines was that of the computational representation of science: how we translate scientific concepts into a language computers can process. Encoding complex scientific entities (e.g., molecules, proteins, and genomes) into computationally tractable formats remains a fundamental bottleneck. This matters because without a standardized, semantically rich language across scales and disciplines, we cannot effectively generate hypotheses, plan experiments, or identify shared patterns across fields (Latent Science; Table 1). Existing projects such as the World Avatar Project are working toward a universal, ontology-driven digital framework that enables the integration and interoperability of complex scientific data across domains.^9^

In experimentation, several challenges proved similarly universal. First, computational cost remains one of the largest limitations for the choice of methodology. Although AI promises to decrease the cost of accuracy, tackling “intractable” problems likely requires new computational paradigms beyond simply scaling traditional architectures. Second, for machine learning models, a recurring issue is the failure to generalize outside their training domain, and without robust real-world benchmarks, it is difficult to know whether a model is optimizing for meaningful signals or artifacts. Researchers often attribute this behavior to insufficient training data. However, the challenge also encompasses the quality of data (DataRaccoon; Table 1), which is affected by selection and publication biases, and the utilization of existing data. We agreed that we must encourage innovation with the data we have, in parallel with generating more. A key insight from the natural sciences is that discovery often happens precisely when generalization breaks down. Therefore, studying failure points of models, AI or otherwise, could be a powerful method for uncovering new phenomena. To evaluate to what extent current AI tools can do this, we propose benchmarking by training on historic data and testing whether they can rediscover subsequent known breakthroughs (Fossilised LLMs, Table 1), which could be compared to previous work on the evolution of knowledge networks.^10^ Third*,* the mismatch between computational predictions and laboratory timescales can confound progress, making it difficult to measure real-world model performance (Protocompare, SupplyScout; Table 1). To further tighten the computational-experimental loop, we propose advanced sensor platforms and cross-disciplinary fellowships to close practical and cultural gaps between computational and experimental labs (Advanced Sensor Platforms, Cross-Disciplinary Fellowships; Table 1). Finally, reproducibility is a shared pain point,^11^ often stemming from inconsistent version control and missing crucial experimental steps (ForGit About It!, GreatProtocols; Table 1).

Across our hackathons, four prototypes were awarded prizes. Protocompare won the community vote, chosen by participants as the tool they would most want to use in their own research. It addresses the problem that many protocols exist for the same procedure, whether in the wet lab or for computational pipelines, but researchers lack a systematic way to compare them. Protocompare aggregates them, breaks them into comparable steps, assesses their similarity, and ranks them using field-relevant metrics such as safety, cost, and computational expense. This enables more informed method selection and more meaningful comparisons between papers with slightly different methodologies, where the impact or extent of those differences might otherwise be unclear.

The remaining winning projects were GreatProtocols, ELIE, and ForGit About It!. GreatProtocols aims to tackle the problem that crucial experimental details are often lost. It prompts the researcher with targeted questions to capture missing information and metadata, building a more complete record of what was done. This detailed protocol can then be regenerated and tailored to the reader’s experience level: a new student receives a thorough walkthrough, while a senior student receives a more concise summary. ELIE helps researchers understand concepts outside of their own domain. The user inputs a target concept, and a graph of prerequisite knowledge is generated. The user then indicates which concepts they already understand, and the process repeats until the boundaries of their existing knowledge are mapped. A tailored explanation is then generated that only expands on the user’s actual gaps. Finally, ForGit About It! addresses the problem of inconsistent version control by monitoring a codebase and automatically generating meaningful commit messages when it deems a logical unit of work to be complete. At the end of the day, it produces a summary of progress made and tasks still remaining.

All hackathon projects are proof-of-concept prototypes, not polished tools. Their value lies in demonstrating what becomes possible when researchers from different disciplines converge on shared problems, and in seeding ideas that can be developed further.

We also discussed the potential impact of AI across our fields. Although many are excited by the promise of AI to address some of the challenges outlined here, the growing adoption of AI tools presents a risk of corrupting the scientific record. In exploration, AI hallucinations can steer fields toward nonexistent phenomena. In experimentation, AI-generated code, analysis pipelines, and papers can contain subtle errors that silently corrupt results at scale. As researchers grow complacent with AI assistance, the risk of unchecked outputs grows. We advocate for proactive measures: accountability, interpretability, and uncertainty quantification must be built into AI tools from inception. Although this slows deployment, we believe it is nonnegotiable for preserving scientific integrity. We also worry that large language models (LLMs) may inadvertently deepen disciplinary silos; if researchers rely on generative AI for a “good enough” answer, they may bypass the deep, necessary conversations with colleagues in other fields. Indeed, although AI appears to increase the pace of producing research outputs, it may narrow their scope.^12^

Beyond the hackathons, CompMotifs has built a lasting community. A WhatsApp group of over 70 researchers continues to share events, funding opportunities, and research outputs. Our LinkedIn presence has grown to around 150 followers. The experience has changed how we all work. For instance, one author who works in a wet biology lab has since developed their own Python tool for analyzing experimental data, directly inspired by exposure to computational approaches they would not otherwise have encountered. Several authors have used ELIE in their own research to understand concepts outside their domain. These are modest examples, but they illustrate how cross-disciplinary exposure can shift individual practice.

The acceleration of science using computation requires more than larger datasets or faster hardware; it demands a cultural and conceptual shift. We propose four actionable recommendations to accelerate scientific progress. First: *fund interdisciplinary micro-communities and hackathons*. Small, agile groups like CompMotifs and hackathons can bring together researchers who would not otherwise interact, and produce prototype solutions. Second: *incentivize a culture of openness*. Bringing together computational and experimental researchers and focusing on scientific value rather than commercial potential created conditions where shared problems could be identified and addressed. Third: *establish a “data lake” of the scientific process*. We need to log the full scientific process, including failures and detailed protocols, not just the polished outcomes, for transparency and reproducibility and to be able to train genuinely useful AI co-scientists. Fourth: *develop robust, cross-disciplinary benchmarks* that reflect real-world goals to track genuine progress and prevent gaming of metrics.

By investing in interdisciplinary communities, we can break down the silos that stifle innovation. Take our prototypes and ideas, develop them, critique them, build upon them, or create something entirely new. Run your own hackathon. The ultimate goal is a transparent and efficient ecosystem where cross-pollination of ideas happens naturally and unlocks a new scale of shared scientific discovery.

## Acknowledgments

Funding was provided by the Advanced Research and Invention Agency (ARIA) through the Innovator Circles program. We thank all speakers for their time and insights and hackathon participants for their enthusiasm and prototypes. F.T.S. thanks the Royal Society for a Royal Society University Research Fellowship. A.M.M. is supported by the Eric and Wendy Schmidt AI in Science Postdoctoral Fellowship, a Schmidt Sciences program. We thank Tomasz K. Piskorz for his careful review of the manuscript and for his valuable comments and suggestions.

## Declaration of interests

J.A. and D.A. are cofounders of Callosum Technologies. K.D. is an employee of NVIDIA. P.R. is a cofounder of Geodesic Research.

## Declaration of generative AI and AI-assisted technologies in the writing process

During the preparation of this work the authors used Gemini, ChatGPT, and Claude in order to write the first draft based on an outline and discussion notes. After using these tools, the authors reviewed, substantially edited, and rewrote the content as needed, and they take full responsibility for the content of the published article.
